# Prepartum Maternal Behavior of Domesticated Cattle: A Comparison with Managed, Feral, and Wild Ungulates

**DOI:** 10.3389/fvets.2018.00045

**Published:** 2018-03-12

**Authors:** Maria Vilain Rørvang, Birte L. Nielsen, Mette S. Herskin, Margit Bak Jensen

**Affiliations:** ^1^Department of Animal Science, Aarhus University, Tjele, Denmark; ^2^INRA, NeuroBiologie de l’Olfaction, Université Paris-Saclay, Jouy-en-Josas, France; ^3^INRA, Modélisation Systémique Appliquée aux Ruminants, AgroParisTech, Université Paris-Saclay, Paris, France

**Keywords:** behavioral plasticity, birth place, cattle, isolation seeking, maternal behavior, motivation, olfaction, parturition

## Abstract

The event of giving birth is an essential part of animal production. In dairy cattle production, there are substantial economical and welfare-related challenges arising around the time of parturition, and hence increased focus on efficient management of the calving cow. Drawing on the research literature on prepartum maternal behavior, this review compares cattle to other members of the ungulate clade with the aim of understanding the biological basis of bovine prepartum behavior with main emphasis on dairy cows. Ultimately, this knowledge may be used in future development of housing systems and recommendations for the management of calving cows. Maternal prepartum behavior varies among species, but the final goal of ungulate mothers is the same: ensuring a calm parturition and optimal environment for the onset of postpartum maternal behavior by locating an appropriate birth site, with low risk of predators, disturbances and mistaken identity of offspring. Features of chosen birth sites vary among species and depend largely on the environment, as ungulate females display a considerable ability to adapt to their surroundings. However, within commercial housing conditions in dairy production, the animals’ ability to adapt behaviorally appears to be challenged. Confinement alongside high stocking densities leave little room to express birth-site selection behavior, posing a high risk of agonistic social behavior, disturbances, and mismothering, as well as exposure to olfactory cues influencing both prepartum and postpartum maternal behavior. Dairy cows are thus exposed to several factors in a commercial calving environment, which may thwart their maternal motivations and influence their behavior. In addition, prepartum cattle may be more affected by olfactory cues than other ungulate species (e.g., sheep) because they are attracted to birth fluids already before calving. Hence, providing dairy cows with an environment where they can perform the maternal behavior they are motivated for, may aid a calm and secure calving and provide optimal surroundings for postpartum maternal behavior. Future research should focus on designing motivation-based housing systems allowing freedom to express prepartum maternal behavior and investigate in more detail the effects of the environment on the welfare of calving cows and their offspring.

## Introduction

The event of giving birth is an essential part of animal production. There are substantial economical and welfare-related challenges arising around the time of parturition, and commercial animal production have developed an extensive body of recommendations for housing and managing parturient females. In beef and dairy production, successful management of the calving cow aims to ensure a viable calf with no detrimental effects for the cow. In addition, a smooth transition from dry to lactating is important for dairy cows. To achieve these goals, recommendations state that careful supervision during calving and timely intervention is crucial. Hence, calving cows should be kept in a way that enables the farmer to identify cows in need of assistance. Recent guidelines suggest that cows should calve in individual pens [e.g., by law in Denmark (1) and in The Canadian Dairy Code of Practice (2)] partly based on the finding that cows increase the distance to the herd before calving if they have the opportunity (3). These guidelines appear to be well suited to the behavior of parturient cows, but the motivation underlying this behavior is not known. Are the cows motivated to move away from the herd to avoid other cows, to hide from disturbances in general, or are they attempting to hide from specific threats? If the causal factors underlying the prepartum behavior of parturient cows are not understood, is it then certain that aspects of animal welfare related to behavioral needs and highly motivated behavior are accounted for when cows are kept in individual pens at calving? Keeping cows in individual pens benefit the farmers—and by extension health aspects of dairy cow welfare—due to easier calving supervision and assistance when needed, but does it also satisfy maternal motivation of the cows?

**Figure 1:**
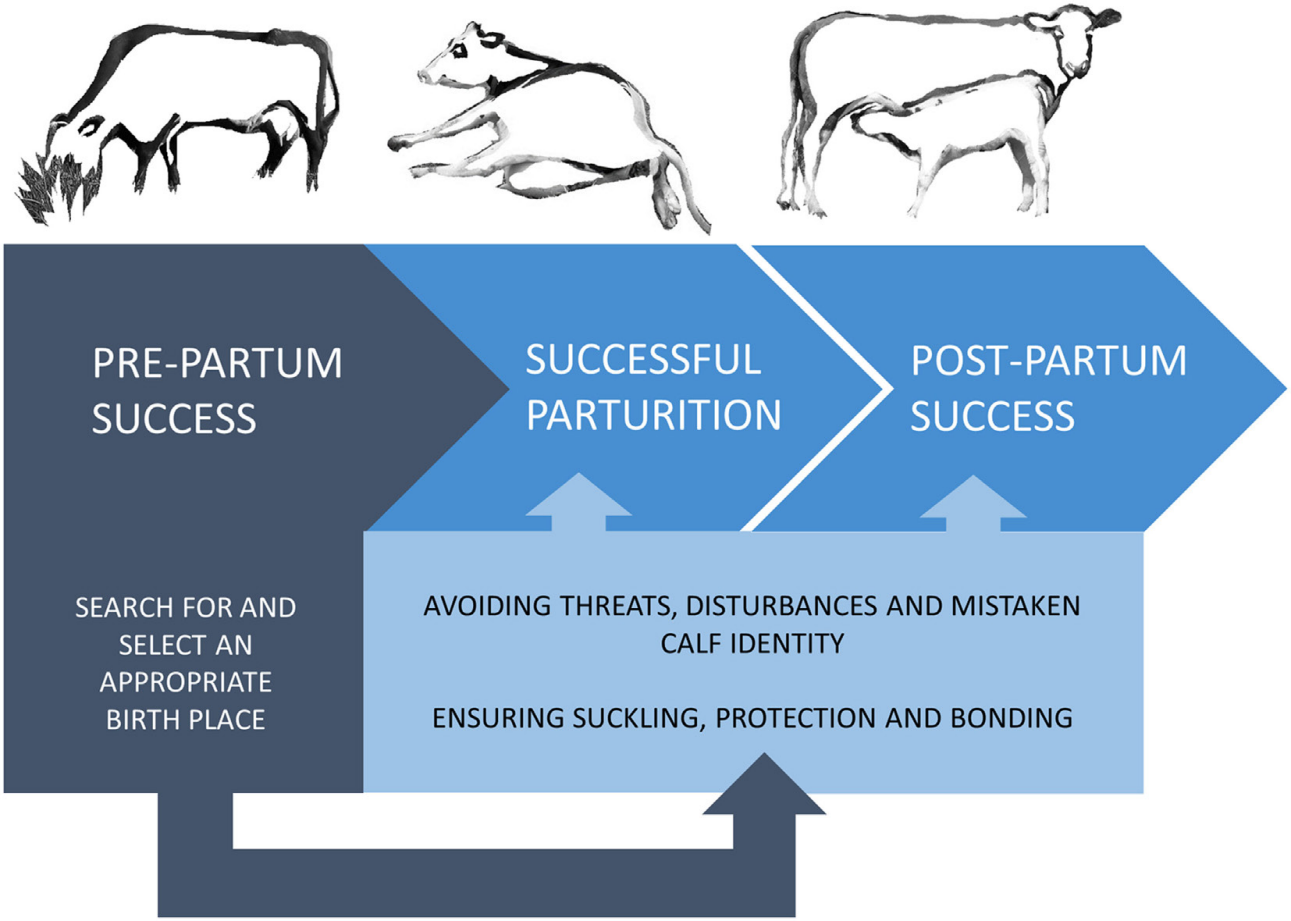
The impact of the prepartum search for and selection of an appropriate birth site. Prepartum success depends on the female’s ability to locate an appropriate birth site to ensure and safeguard a calm parturition and optimal surroundings for postpartum maternal behavior by lowering the risk of predators, disturbances, and mistaken identity of offspring. This, in turn, increases the chance of postpartum success.

The survival and development of mammalian young depends largely on a strong mother–offspring relationship. The clade Ungulata includes mainly precocial species giving birth to well-developed offspring, capable of moving on their own shortly after birth (4). To protect their vigorous offspring, ungulate mothers exhibit complex behavioral patterns starting in late pregnancy and continuing through parturition and lactation (5). This differs substantially from the normal adult female behavior and functions to provide the young with sufficient nutrition, warmth, protection, comfort, and opportunities for social transmission of information [as reviewed in Ref. (6)]. In the domesticated species, reproductive success has a huge impact on productivity, and thus scientific focus has been mainly on successful parturition and subsequent lactation, and far less on the period leading up to parturition. Both beef and dairy cattle production rely on the cows’ ability to reproduce, but it is only in beef cattle production that farmers depend on the ability of the cow to establish a strong and long-lasting bond to her calf, providing it with nutrition and protection until weaning (7). Dairy production is based on the cow’s ability to produce milk after removal of the newborn calf (7), and thus selection for maternal behavior in dairy cows may have been relaxed compared with beef cattle due to the reduced need for post-calving maternal investment. However, this does not take into account the inevitable need for prepartum maternal behavior aiming to ensure smooth calvings with few (or preferably no) complications. This will further safeguard strong and healthy calves, as well as healthier and more productive cows with low morbidity (Figure 1). Indeed, post-calving success is likely to be dependent on the pre-calving success, which emphasizes the need for appropriate prepartum maternal behavior.

A small body of literature has shed light on the prepartum maternal behavior of the cow, although mainly under production conditions [e.g., Ref. (8, 9)] and only to a lesser extent under semi-natural conditions [e.g., Ref. (3, 10) further details Table 1]. To date, there are only few studies on the prepartum maternal behavior of feral cattle [Maremma cattle (11), Masai cattle (12), Chillingham cattle (13), and Camargue cattle (14)] and these were all carried out several decades ago. This may seem surprising as no less than four articles over the last 40 years have pointed out the need for more comparative studies of ancestral and domestic behavior in cattle (15–18). Given that the ancestor of cattle, the Auroch, has been extinct for centuries (19) and the number of feral cattle herds are very limited (Table 1), one potential approach to understanding the biology underlying prepartum maternal behavior of domesticated cattle, is by comparison with other ungulate species. Studies of feral cattle may be more likely in the future with the advent of conservation grazing [e.g., Ref. (20)], giving more opportunity to observe prepartum behavior under natural or low-managed conditions.

**Table 1:** Overview of observations within studies of maternal behavior in cattle with main emphasis on prepartum behavior.

	Feral cattle	Pasture-kept cattle	Cattle housed in intensive commercial environment (mainly indoors)
**Features of the birth site**			

Vegetative/visual cover	Differs with habitat^h,k^	Differs with habitat^E^ Mainly visual cover^A^	No clear preference^19^

Provides cover from disturbances	*Not studied*	Yes, from herd members^A^	Calving when quiet in the barn^1,5,21^ Higher stocking density results in lower “isolation seeking”^17^

Distance to herd	Leave the herd but no defined distance^e,g,h,i^ 10–380 m away from the herd^d^	Leave the herd but no defined distance^G^	*Not studied*

**Prepartum behavioral changes**			

Separation from herd	Yes^a,c, e,g,h,i,j^ Only some cows do^b,d,f^	Yes^A,C,E^ Only some cows do^F^ No^B,D,H^	Yes^4,16^ Only some cows do^6^ Yes, but depends on calving difficulty^19^

Restlessness	Yes^d^	Yes^A,C,F^	Yes^1,3,4,10,12,21^ Varies with calving difficulty^2,19^

Increased walking/searching	Yes^d^	Yes^C,F^	Yes^1,7,12,13,14,19,22^

Lying time	*Not studied*	Unchanged^G^	Lower on the day of calving^10,15^ Higher 8 h before calving^3^

Increased transition from standing to lying and *vice versa*	Yes^d^	Yes^A,C,G^	Yes^1,2,3,8,10,12,13,14,15,19,22^

Increased sniffing/exploration	*Not studied*	*Not studied*	Yes^1,14,19,22^ No^2^

Increased tail raising	*Not studied*	Yes^C,F^	Yes^2,11,12,13,14^

Licking own body and attention toward abdomen	Yes^d^	Yes^C^	Yes^1,10,12,22^No^11^

Scraping or pawing the ground	Yes^d^	Yes^C^	Yes^1,22^

Less feeding behavior	Yes^d^	*Not studied*	Yes^10,12,13,14^

Reduced rumination	*Not studied*	*Not studied*	Yes^1,3,7^

**A role of olfaction**			

Licking of own birth fluids	Yes^d^	Yes^A,C,F^	Yes^1,12,14,18,20,22^

Calving at own birth fluid spot	*Not studied*	Yes^A,C^	Yes^18,20^

Mismothering	Not observed^^j^^	Yes^A,B,F^ Not observed^G^	Yes^6,9,19^

Interest and sniffing from other cows during calving	No^j^ Yes^f^ Only from cows close to calving themselves^d^	Yes^A,B,F^ No^G^	Yes^6,9,15,18,19^

*The table includes 41 studies separated into the categories: feral (*n* = 11), pasture-kept (*n* = 8), and intensive commercial cattle mainly housed indoors (*n* = 22). Aspects of and behaviors related to three different subjects (features of the birth site, prepartum behavioral changes, and the role of olfaction) are listed. Numbers and characters in superscript indicate the corresponding reference listed at the bottom of the table, with the number in brackets after each reference indicating the order in the reference list. ‘*Not studied*’ refers to the authors being unable to find any literature on this specific aspect*.

*References: ^a^Baskin and Stepanov (21); ^b^Finger et al. (22); ^c^Hall (13); ^d^Kiley-Worthington and de la Plain (23); ^e^Lent (4); ^f^Lidfors and Jensen (24); ^g^Reinhardt (25); ^h^Reinhardt et al. (12); ^i^Schloeth (14); ^j^Vitale et al. (11); ^A^Aitken et al. (26); ^B^Edwards (27); ^C^George and Barger (10); ^D^Lidfors (28); ^E^Lidfors et al. (3); ^F^Owens et al. (29); ^G^Rice et al. (30); ^H^Wood-Gush et al. (31); ^1^Arthur (32); ^2^Barrier et al. (33); ^3^Borchers et al. (34); ^4^Dufty (35); ^5^Edwards (36); ^6^Edwards and Broom (16); ^7^Houwing et al. (17); ^8^Huzzey et al. (37); ^9^Illmann and Špinka (38); ^10^Jensen (8); ^11^Lange et al. (39); ^12^Metz and Metz (40); ^13^Miedema et al. (41); ^14^Miedema et al. (42); ^15^Proudfoot et al. (43); ^16^Proudfoot et al. (44); ^17^Proudfoot et al. (45); ^18^Rørvang et al. (46); ^19^Rørvang et al. (47); ^20^Selman et al. (48); ^21^von Keyserlingk and Weary (49); ^22^Wehrend et al. (9)*.

This review draws on literature from feral and commercial cattle breeds and investigates similarities and dissimilarities to other members of the ungulate clade. With the main emphasis on dairy cows, our aim was to understand the biological basis of prepartum behavior of feral cattle to improve the understanding of motivations underlying and mechanisms causing the behavior seen in domestic cattle today. In the future, this knowledge may benefit the dairy industry and lead to better-adapted housing system designs and recommendations for better prepartum management practice, which improves both efficiency and animal welfare.

## Why Isolate?

Many ungulate studies have reported that a proportion of the females are “hiding,” “isolating,” “being secluded,” or “seeking away” from the herd or from other “threats” around the time of parturition. The term “isolation seeking” is commonly used in such studies, but what is termed isolation in one species may differ from what is termed isolation in other species. Irrespectively, the term “isolation seeking” is used to indicate the purpose of the behavior: to hide and seclude the female from disturbances (arising from various threats), thus allowing her to give birth in a calm place, where she subsequently is able to nurse and bond with her young. However, as isolation seeking in one ungulate species may differ from that of other species, the comparison of different ways to achieve the same goal is relevant, especially as the underlying motivations of females of different species may or may not be the same. In the following, isolation behavior is discussed in the context of causality, whereas the hider/follower paradigm is dealt with in Section “The Hider/Follower Paradigm,” although some overlap is unavoidable.

In sheep, Dwyer and Lawrence (50) suggested that birth-site selection (termed “isolation seeking”) varies with increasing degree of domestication. Wild and feral breeds of sheep such as mouflons, Soay, Dall, and bighorn are observed to move away from the herd to rocky and secluded areas (51–55), hence they may not distance themselves from the herd, but seek cover, or a combination of the two. Domesticated breeds such as Merino sheep also distance themselves from the herd, but only when the environment offers a degree of elevation or topographical change, otherwise the ewes give birth within the herd (56). This may be influenced by artificial selection for more sociability in the domestic breeds (50), but is evidently also affected by the environment (see The Hider/Follower Paradigm).

So far, some ungulate studies have sought to explain prepartum isolation seeking behavior of females, and noted that the characteristics of the birth site itself may be less important than the ability to move away from disturbances (57). An example of this can be seen in wild Thomson’s gazelles (58): Roberts and Rubenstein showed that if a herd caught up with a parturient female, the newborn fawns were usually killed by jackals, presumably due to the group being too conspicuous. In such cases, being disturbed by the herd can have fatal consequences, and since disturbances during parturition were much more common for non-isolating than for isolating females, the hiding aspect of the isolation seeking behavior appears important for the survival of the offspring in this species. Yet, other studies indicate that fallow deer dams adapt their maternal behavior to the prevailing predator pressure, which even may supersede forage availability (59). This is a sensible priority as a predator is an acute survival threat as opposed to lack of food, which may be tolerated in the short term. Another example of prepartum isolation comes from breeds of domestic sheep, which move to the edges of their enclosure to lamb, thought to be caused by disturbances arising from human activity (50). Likewise, indoor-housed domestic sheep will use a cubicle at lambing when given the opportunity (60, 61). In addition, when disturbed by human activity in the area, elk dams will change their movement pattern, especially if disturbed during calving season (62). Assuming that wild ungulate dams perceive humans as predators, such behavioral flexibility, may have originated from sensitivity to predator pressure (58). Avoiding predators by hiding is an adaptive behavior as it reduces the risk of having the offspring killed and these behaviors may thus be preserved in domestic species. Hence, isolation may be a means to avoid disturbances in general, but more specifically avoid predators or other immediate threats. Irrespectively, in a commercial livestock production environment, where females are surrounded by herd mates, hiding will often be difficult, especially if human activity and other disturbances are frequent. More work is needed to examine whether domestic females are aiming to avoid threats, and whether disturbance may cause artificial isolation opportunities to be less attractive [as suggested by Rørvang et al. (63)]. The level of disturbances can be high in commercial environments (i.e., from humans and conspecifics), and the use of an artificial hide by the cow may reduce her perceived ability to escape a potential threat; hence, some artificial hides may not provide an attractive birth site.

Another adaptive aspect underlying isolation is a reduced risk of mismothering, i.e., cows licking and nursing calves that are not their own offspring. The immediate licking and sniffing of the young by the dam are part of the typical behavioral repertoire of ungulates enabling the mother to learn the odor and features of her young for later recognition, thereby ensuring that her parental investment is directed toward her own offspring (54, 64). Several studies in domestic cattle have shown that group housed peri-parturient cows may lick alien calves, i.e., calves born from other cows [e.g., Ref. (27)] and cross-fostering (i.e., when a cow adopts an alien calf by allowing it to suckle) has also been reported [e.g., Ref. (65)]. However, observations from feral cattle herds indicate that cows rarely nurse or lick alien calves [Maremma cattle (11), Masai Cattle (12)]. The feral cow and calf may develop a stronger mother–offspring bond, which is established through intensive contact during a sensitive period just after parturition [potentially just a few hours after calving (4, 49)]. This bond may be established quicker in an undisturbed calving environment, which may not be available for domestic cows in a group pen. Calving in a group pen leads to an increased risk of mismothering and failure to obtain colostrum by the calf, thereby challenging the transfer of immunity *via* colostrum intake (27, 38). One reason for the observed mismatches between dairy cows and alien calves may be a weakening of the maternal motivation in dairy breeds. Even though dairy cow maternal behavior may have been modified by genetic change, studies from other domesticated animals [e.g., nest building in pigs (66, 67) and mice (68)] suggest that maternal behavior is preserved despite domestication. Although we cannot exclude that the occurrence of mismothering reported by studies on dairy cattle to some extent is affected by genetic change, a more likely influential factor is disturbances caused by the confined environment. Taken together, the above comparison of prepartum maternal behavior of female ungulates suggest that the behavior described as isolation seeking may be an expression of birth-site selection; functioning to safeguard a calm and secure birth process by avoiding threats and disturbances potentially posing a risk to the survival of the female and the newborn in terms of predation and mismothering whilst at the same time ensuring suckling, bonding, and protection (Figure 1).

## What are the Properties of an Appropriate Birth Site?

Natural selection favors mothers that display behavior and habitat selection to enhance neonatal survival (59, 69, 70). Hence, in a variable environment, natural selection will favor mothers that are able to modulate and adapt their maternal behavior including habitat selection to the prevailing circumstances. This ability to adapt is evident in an array of maternal behaviors. For example, if ungulates are kept in environments with few options to search actively for an appropriate birth site, the searching behavior displayed by the females may be less pronounced. Due to the scarcity of dairy cow studies on these issues, this section will draw predominately on findings from other ungulate species. Fouda et al. (71) reported that zoo-kept sika deer, a species known to hide their offspring in nature, gave birth within the herd. The authors concluded that this behavior resulted from the lack of suitable sites where a fawn could be hidden. Lott and Galland (72) saw some isolation seeking in American pasture-kept bison. They stated that the bison gave birth away from the herd when vegetative cover offered visual isolation from the herd, whereas calving happened within the herd when visual isolation was not possible. Roberts and Rubenstein (58) found that Thomson’s gazelle females spent considerable time searching for a suitable place to give birth (sometimes traveling more than a kilometer) and mainly gave birth in tall grass away from the herd. However, the authors observed that a herd would occasionally catch up with the parturient female, negating the effects of cover availability by their presence. For other ungulates, clear topographical birth-site preferences have been found. Feral goats appear to prefer birth sites protected by an overhead or vertical cover, e.g., trees or hedges (73). Domestic sheep are known to predominantly give birth on slopes and in depressions in the ground or areas close to hedges and walls (74, 75), whereas mountain sheep are attracted to high, rocky areas with cliffs (51, 76). Other species, such as red and fallow deer (59, 77, 78), pronghorn (79), elk (80), wild mouflon sheep (52), and moose (81), favor thick vegetative cover providing visual isolation from conspecifics. For these species, further studies are needed to ascertain if such preferences are expressions of motivation to isolate in terms of distance from the herd or to hide from the herd as well as other disturbances including predators. Cattle do not appear to show clear preferences for specific birth-site types, even though a few studies on dairy cows have tried, without success, to elucidate what features are favored (27, 47). Across studies of bovine birth-site selection, the presence of vegetative cover may play a role (3, 11, 24, 26) for the occurrence of isolation behavior.

## Does Separation Distance from the Herd Matter?

One important aspect of birth-site selection is the physical distance the parturient female moves away from the herd. In many ungulate species, parturient females distance themselves from the herd [zebra (82); sable antelope (83); bison (72); elk (80); pronghorn (79); horse (84); red deer (77, 85); impala (86); goat (87); various wild sheep breeds (51, 53, 76, 88, 89)], although the exact distance moved by the females has received only modest attention. The only mention of this was by Karsch et al. (76), who found that parturient ewes of wild breeds moved more than 2 km away from the herd. Many studies included distance from the herd as part of the definition of isolation when studying prepartum behavior of females, but only rarely noted the actual distance. For example, Kiley-Worthington and de la Plain (23) observed free-ranging cattle and noted that isolation seeking was rare even though they did not include a definition of the term other than observing cows moving 10–380 m away from the herd. The authors also noted that the herd sometimes moved with the pre-parturient cows, thereby reducing the distance between them, similar to the findings by Roberts and Rubenstein in Thomson’s gazelles (58). Another study by Flörcke and Grandin (90) found that red angus beef cows moved 25–1,250 m away from the main herd when calving and the authors further noted that 88% moved more than 100 m away. One complicating aspect of distance between the parturient female and potential threats or disturbances in her environment is the interaction between the distance and the possibility to hide. For example, when ungulates live in flat and barren environments, hiding the offspring becomes difficult irrespective of the maintained distance to threats/disturbances. Blank et al. (91) found that for goitered gazelles in a habitat without vegetative cover, where the mothers were unable to visually hide their offspring, distance between mothers and offspring became crucial for the mother not to attract predators to the young. As the mother was unable to visually hide the offspring, she increased the distance to the offspring, seemingly to compensate for the lack of vegetative cover. This was shown in pronghorn mothers living on mixed-grass prairies, where the mothers separated themselves on average 269 m from the young (92). In other words, these mothers distanced themselves to where the young was hiding when cover was deficient indicating that the increased distance was motivated by protection of offspring from predators. Unfortunately, no data are available for domesticated ungulates kept under natural conditions, but the above findings suggest that cover is an important part of birth-site selection, and that parturient females only relocate long distances in situations where physical cover is limited.

Although isolation, hiding, seclusion, and seeking away can all be part of prepartum behavior of ungulates, it may be more appropriate to use “birth-site selection” to describe the ultimate (functional) causation of the behavior observed. Female ungulates appear to favor birth sites providing protection from predators as well as conspecifics, and the preferences of the dams seem largely to depend on the environment. During the selection of a birth site, physical cover may be an important factor, but in situations where such cover is limited, distance from the herd may become increasingly important.

## The Hider/Follower Paradigm

Within ungulate species, two different peri-parturient types are described in the literature; these are the “hider” and “follower” strategies of ungulate offspring and mothers (4, 51, 87, 93). However, comparative research within ungulate species has shown that the hider–follower dichotomy may be overly simplistic, and that a number of species may be either, depending on the circumstances. Thus, instead of being either/or, in reality, hiding and following strategies may form part of a continuum, and both of these are considered antipredator strategies. Hiders provide protection in terms of hiding the young in covered or secluded habitats after giving birth (4), while followers actively look out for and avoid predation in open habitats by keeping their offspring close (4, 93). Incorporated into the continuum of these two behavioral types is the dependence on the environment, and at present little is known about the extent to which the behavior of an *individual* ungulate mother and young varies depending on variations in the environment.

It is suggested (87) that goats, which are considered typical hider species, were only able to express “true hider characteristics” when kept in their natural environment. In accordance, Tennessen and Hudson (94) found that in domestic goats, early mother–kid contact shared more characteristics with the behavior of follower species. These authors suggested that either the hider characteristics of goats were lost through domestication or maternal behavior changed when the animals were kept in a different environment. Later, studies of goat behavior showed that domestic goats do separate themselves from herd mates before kidding (95). Also, their rather complex hider behavior appears to be largely genetic, making it a highly motivated behavior and thus less prone to evolutionary dilution (96), even though it may be influenced by environmental factors.

Feral populations of ancient cattle breeds living in large and non-managed nature reserves may provide insight into the maternal behavior of non-domesticated cattle. Cows from African and Camargue cattle herds have been observed leaving the herd days or hours before parturition (4, 12, 14, 25). Calves of Chillingham cattle hide after birth (97), whereas calves of Maremma cattle exhibit both hiding and following behavior in the early weeks of life depending on the availability of cover (11). Similarly, studies in domestic cattle seem to support the above suggestion of a lack of a strict hider/follower dichotomy. There are reports of cattle seeking away from the herd before birth when kept in large, open, and non-managed natural environments (4, 11–14, 21, 25), when pasture-kept (3, 10, 26) and when housed under commercial production conditions (44), but many studies report only some cows or no cows separating themselves from their herd mates (Table 1). As with sheep, the studies listed in Table 1 indicate that prepartum separation is more common in feral types of cattle, whereas studies of pasture-kept or indoor-housed cattle rarely report such behavior. This may be due to domestication favoring less fearful, more social animals, which are more stressed by social isolation as suggested for sheep (50) or, perhaps more likely, due to the confined environment in which the animals are usually kept. There is not enough evidence to suggest that domestic cattle display different intermediates of hider and follower strategies although cattle may adapt to the environment they inhabit.

## Prepartum Behavior

In wild ungulate species, only few observations on female prepartum behavior have been recorded (as opposed to postpartum behavior studies), which may be caused by the animals not being present near the herd around parturition. Within studies of ungulates kept under commercial housing conditions, most authors describe some of the following behavioral changes occurring as parturition approaches: pacing, pawing, circle walking without an obvious goal, frequent postural changes, and reduced lying duration [domestic goat (96), domestic sheep (98–100), and red deer (85)]. In cattle, similar prepartum behavioral changes have been described (Table 1). Restlessness is the behavior most often reported in cows when calving is imminent (Table 1). There is, however, a discrepancy in the interpretation of the described restlessness: is it caused by motivation to search for an appropriate birth site, the experience of pain, or is it a sign of frustration? The causation for the restless behavior prepartum and during labor is currently not fully understood. The process of giving birth is most likely painful (101), and pain may therefore be involved in the behavioral changes prepartum. The behaviors observed at this time (reduced lying, increased walking, walking with no obvious goal, reduced eating, pawing, pacing along fences, and more frequent posture changes; Table 1) are all often interpreted as signs of restlessness and thus the definition of restlessness varies considerably between studies. So, even though these behaviors may all reflect the same motivation of locating an appropriate birth site, the constraints of the confined environment may cause the restless behavior. Similar arguments have been put forward by Wass et al. (85), who suggested that fence line pacing in pre-parturient red deer may be a result of the hind being thwarted in searching for and locating an appropriate birth site. Other authors have suggested that high stocking density or low inter-individual distance may cause pacing or restlessness due to the inability of the female to distance herself from the herd (85, 98). Studies quantifying prepartum behavioral changes in cattle with the aim of predicting calving time have failed to identify a specific type of behavior, which reliably predicts the timing of calving [e.g., Ref. (34, 102)]. However, it is agreed that a combination of several behavioral indicators improves estimation of calving time, as any one behavioral indicator cannot reliably predict time of calving [e.g., lack of rumination 70% in sensitivity and specificity (103)]. One possible explanation for these findings may be that all behavioral changes at this time are affected by the same underlying motivation, or thwarted motivation, to search for and find an appropriate birth site. If so, the apparent absence of reliable behavioral indicators may reflect different attempts to adapt to the situation, which depends on environmental factors (8, 30, 37, 42, 104, 105).

Focusing on lying behavior, Huzzey et al. (37) measured frequency and duration of standing in cows kept in individual pens on the day of calving, and compared this with the behavior of the same cows before and after calving, when they were group housed in free-stalls. Stocking density remained the same (one cow per stall), but the environment changed markedly on the day of calving, i.e., from group to individual housing. The authors found reduced lying time corresponding to an approximately 2 h reduction, and 80% more standing bouts on the day of calving. Jensen (8) and Miedema et al. (42) also found reductions in lying time on the day of calving (1.3 and 1 h, respectively), as well as increased frequency of lying bouts in the last 6 h before calving. In these studies, the cows had more time (i.e., several days) to adjust to the environment before calving than the cows studied by Huzzey et al. (37) (which had 24 h or less). In contrast to these findings from indoor calving studies, a recent study by Rice et al. (30) found no reduction in lying time and only an increase in lying bouts 3–4 h before calving in cows calving on large pasture. Therefore, it is possible that the behavioral responses observed as calving approaches are signs of failed behavioral attempts to adapt to the confined environment. If so, restlessness may be a sign of frustration resulting from the cow not being able to search for and find an appropriate birth site, rather than a sign of stress or pain induced by parturition *per se*. One might argue that as birth-site selection behavior is observed in large and open environments, restlessness may be seen in the confined environment because the calving cow moves as if she was in the large environment. The behavior is similar, but the environment affects its expression and hence its interpretation.

Frustration from being prevented from performing prepartum maternal behavior has been documented in at least one ungulate, the domestic pig. Crating of parturient sows, as is typically done in commercial housing systems, prevents the choice of nesting site [feral sows will walk kilometers to choose an appropriate nesting site (66)] and prevents the performance of natural prepartum nest building mainly due to lack of space and lack of nesting materials (106, 107). The higher activity level measured pre-farrowing, such as frequent changes between standing and lying (108, 109), is most likely a reflection of the inability to search for a nesting site. Abnormal behaviors such as bar biting (110–112), rooting the floor, and sham chewing (111, 113) are also seen in the period leading up to farrowing. Moreover, loose housed sows provided with pre-formed nests still perform nest building behavior (114) and thus achieving the goal of having a nest does not satisfy this behavioral need. The high activity level and the abnormal behaviors may reflect the same underlying cause as the restlessness seen in cattle and many sow studies suggest that these are signs or out-lets of frustration arising from not being able to express the highly motivated prepartum maternal behavior. This view is further supported by the findings that preventing sows from nest building activities results in decreased oxytocin levels (113, 115), increased cortisol concentrations (111, 116), and increased heart rate (117), leading several authors to propose that impairment of natural behavior during the prepartum period results in compromised welfare of sows (111, 117–119). Also, confined sows have longer farrowing durations and longer inter-piglet birth intervals, thereby challenging the vitality of the offspring (107). Such measurements are not available within studies of prepartum behavior of cattle but we do know from work on social isolation and lying deprivation [measured as ACTH increase in Ref. (120)] that non-parturient cows show signs of frustration. In cows, more studies of the consequences of allowing the possibility to perform prepartum maternal behavior are needed to understand the motivational background of the prepartum behavior observed in cows in commercial production systems. Such studies would enable evaluation of whether and when motivation-based systems mitigates the expression of prepartum behavior, thereby improving the welfare of calving cows and their calves.

## A Possible Role for Olfaction?

Olfaction is an aspect of maternal behavior in cattle which has received little scientific attention until now. In many ungulate species, birth fluids are attractive and consumed by parturient females, e.g., domestic and wild sheep (4, 56, 98, 121), horses, pigs and goats (122), sable antelopes (123), and red deer (85), but this behavior has only been studied sparsely in relation to ungulate mothers’ selection of birth site. However, the attractiveness of birth fluids is closely related to parturition. In sheep, the attraction has been shown to last for a few hours after lambing (121), whereas cows show signs of attraction as early as 12 h before calving lasting for at least 24-h postpartum [the duration of the study (124)]. George and Barger (10) found that parturient cows remained within the same area where their amniotic fluids were discharged until calving had been completed, and recently Rørvang et al. (46) suggested that cows predominantly would calve at the spot where another cow had previously calved. Attraction to olfactory cues therefore appears to have implications for the prepartum maternal behavior of cattle. Maternally motivated cows kept in groups are inevitably affected by the odor cues in the birth fluids of other cows even before giving birth themselves, and this may be exacerbated when housing conditions prevent cows from avoiding these odors. In addition, the attractiveness of these odors may reduce the likelihood of a cow moving away to find a birth site elsewhere, which may make artificial hides less attractive (63). Based on the above, we suggest that olfactory cues need to be considered in future prepartum maternal behavior studies and are likely to influence the use of any calving facility provided.

Olfactory cues, however, are not only important for the prepartum behavior of female ungulates. In sheep, the role of olfaction is essential for the onset of lamb-directed maternal behavior, at least for inexperienced mothers (125, 126). For instance, Basiouni and Gonyou (125) showed that fostering of alien lambs to parturient females was possible only if the lambs were covered by jackets soaked in amniotic fluid. Adult domestic goats show interest in alien newborn kids (95), and in farmed red deer such attention can be rather intense and even increase if stocking density is high [in addition, more mismothering occur in this situation (85)]. As mentioned earlier, also cattle studies have reported attention from cows toward and licking of alien calves, especially in commercial housing conditions (Table 1). Studies from free-ranging cattle, however, often do not report cows showing interest in alien calves’, which may be explained by the cows seeking away from the herd (11). Hiding or separating from conspecifics probably lowers the risk of mothers interacting with alien offspring in general, but physical cover may not suffice to keep maternally motivated cows away if they can smell a calf. Recently, we offered pregnant group housed cows an opportunity to select an individual pen as birth site. The presence of a newborn alien calf in the group pen reduced the likelihood of the cows using this opportunity (63), most likely because the newborn calf’s coat contained birth fluids. Hence, olfaction and odors are likely to be important for the onset and direction of maternal behavior also in cattle.

Commercial dairy cow housing conditions often mean high stocking densities in a relatively barren environment offering few options of selecting a birth site away from other cows as well as more disturbances from human activities and conspecifics. Taken together, this means that pre-parturient cows housed in groups are in close proximity to olfactory stimuli important for maternal behavior, i.e., birth fluids from other cows and their calves. Unlike sheep, cattle show a preference for birth fluids also before parturition, and prepartum cows have been reported to nurse alien calves (59, 62), observations which may explain the higher occurrence of mismothering in commercial housing (Table 1). This may also introduce a higher risk of mismothering when cows calve in group pens when compared with parturient sheep, as ewes are not attracted to birth fluids until after parturition (121). In addition, group housing may increase the risk of agonistic social interactions limiting the access of bovine mothers to their own calves. Mismothering and lack of contact between cow–calf increases the risk of colostrum and maternal care being allocated to alien calves, leading to failure of passive transfer of immunity from the mother to her biological offspring. The importance of olfaction and odors thus need to be taken into consideration in the design of housing facilities for parturient cattle (127), especially in relation to group housing. Implications of group housing of calving cows need to be critically addressed as this type of management is quite common [for example, 70% of US dairy operations (128)], and particularly if cows and calves are to remain together post-calving. Keeping parturient cows in groups is normally associated with early cow–calf separation (2) and thus if early calf nursing and cow–calf bonding are to be ensured, housing of parturient cows in individual calving pens appears to be necessary.

## Conclusion and Perspectives

Drawing on research literature on prepartum maternal behavior, this review compared cattle to other members of the ungulate clade with the aim of understanding the biological basis of bovine prepartum behavior with main emphasis on dairy cows. Prepartum success depends on the female’s ability to locate an appropriate birth site to ensure and safeguard a calm parturition and optimal surroundings for postpartum maternal behavior by lowering the risk of predators, disturbances, and mistaken identity of offspring. At present, the motivations of cows underlying the apparent prepartum isolation seeking behavior have not been fully explored. In addition, traditional concepts of ungulate maternal behavior such as the hider/follower-dichotomy appear overly simplistic. Based on the reviewed literature, we suggest that more scientific focus should be given to the prepartum maternal behavior (i.e., the phase of birth-site selection) in dairy cows, as they are exposed to several factors in a commercial calving environment, which may thwart their maternal motivations and influence their behavior and welfare. One such factor is olfactory cues, which may exert stronger effects on prepartum cows than other ungulate species as cows are attracted to birth fluids already before parturition. Providing dairy cows with an environment where they can perform the prepartum maternal behavior for which they are motivated, may facilitate postpartum maternal behavior and success. Further research focusing on motivation-based housing of peri-parturient cows is needed to ascertain the importance of degree of movement and distance from the group within the constraints of dairy housing systems. These studies should include effects on the welfare of calving cows and their offspring. Ultimately, this knowledge may be used in future development of more suitable housing and management systems for calving cows.

## Author Contributions

All authors contributed to the initial idea and early discussions underlying this review. MR did the main literature search and selection and wrote the first draft of the manuscript, including figure and table.

## Conflict of Interest Statement

The authors declare that the research was conducted in the absence of any commercial or financial relationships that could be construed as a potential conflict of interest.
